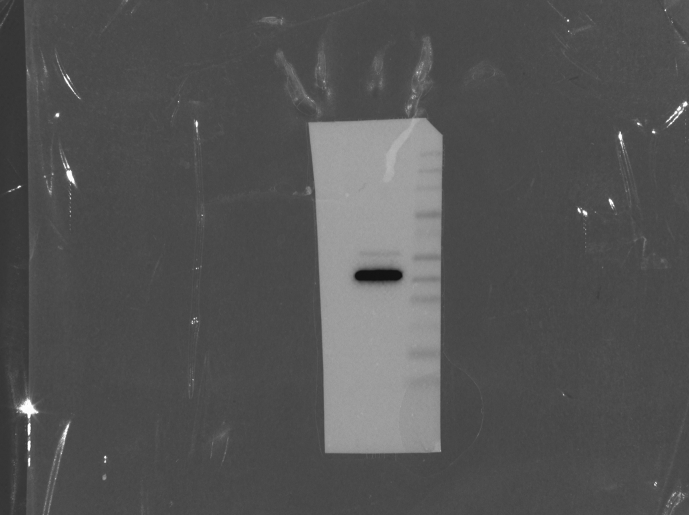# Obtaining source material for cellular agriculture

**DOI:** 10.1016/j.heliyon.2024.e38006

**Published:** 2024-09-17

**Authors:** Apeksha Bharatgiri Goswami, Mark S. Rybchyn, W.R. Walsh, Johannes le Coutre

**Affiliations:** a School of Chemical Engineering, University of New South Wales, Sydney, New South Wales, Sydney, Australia; b School of Clinical Medicine, University of New South Wales, Sydney, New South Wales, 2052, Australia; c Australian Human Rights Institute, University of New South Wales, Sydney, New South Wales, Sydney, Australia

**Keywords:** Cellular agriculture, Cultured meat, Cell line, Bovine, Sustainability, Consumer

## Abstract

Cellular Agriculture (CellAg) is an attractive concept for innovative technology with the intent to provide food and nutrition complementary to existing supply streams. The past decade has seen considerable progress in the field with advancement of cellular technology that delivers the initial building blocks for meaningful implementation. The availability of natural cell-based material that can serve as nutrient-filled source for human consumption at low cost is a critical challenge for the emerging cellular agriculture industry. Therefore, here the isolation of bovine myofibroblasts of the Black Angus breed has been pursued and accomplished together with its characterisation by using RNA sequencing and proteomics through western blotting.

To transition CellAg from a concept to a game changing technology for the industry, multiple challenges need to be overcome. The field requires powerful initial material, i.e., dedicated cells that can proliferate and differentiate robustly at scale. The methodology described allows for the production of healthy cells, which have been unequivocally characterized as clonal representatives of a stable myofibroblast cell line using transcriptomics and proteomics validation. Stringent and rigorous live cell monitoring of a nascent cell line derived from healthy muscle tissue allowed for stable cell growth.

In this research article, a simple and precise methodology is presented for creating a bovine myofibroblast cell line (*Bov.mia*). Our work puts forward a low-tech use of materials and expertise that is devoid of transgenic approaches, thus creating a reliable approach for lab-scale research.

## Highlights

• Presentation of methodology to isolate bovine myofibroblasts for cellular agriculture (CellAg).
• Validation of cell line obtained using RNA sequencing and Proteomics.
• Description of a reliable, non-transgenic approach for CellAg.
• Contextualizing industry needs for stable, nutrient-rich cell sources.

## 1 Introduction

### 1.1 Cellular agriculture technology

CellAg aims to produce agricultural products closely mimicking conventional ones by cultivating them from the cellular level, addressing challenges faced by traditional agriculture in feeding a growing global population, projected to reach 9–11 billion by 2050 [1]. With limited arable land and water, and climate change threats, CellAg emerges as a sustainable alternative, potentially reducing greenhouse gas emissions, land, and water use [2,3]. Traditional livestock farming requires extensive resources and generates significant emissions, with the livestock feed industry accounting for 57 % of total greenhouse gas emissions [4]. CellAg, which involves cultivating tissue samples in controlled environments with nutrient-rich media and bio-scaffolds, could reduce land use by 80 %, water use by 94 %, and greenhouse gas (GHG) emissions by 76 % [5]. Cultured meat should be genetically and cellularly indistinguishable from traditional meat and offers ethical and health benefits by eliminating the need to raise animals for slaughter and reducing zoonotic disease risks [6]. Although in its early stages, CellAg holds promise for consumer acceptance and addresses sustainability and ethical consumption, providing a protein-rich nutrition source without depleting natural resources as the global population expands [7,8].

### 1.2 The emerging industry

Despite extensive research and market interest in CellAg, uncertainty remains about its ability to meet expectations and public reception. Currently, these products are limited to select boutique restaurants. R&D focuses on cell line advancement, media formulation, scaffold design, and production scaling using bioreactors. Around 200 cultured meat startups worldwide reflect the belief that "animal-free" meat addresses an unmet consumer need. Initially, companies pursued direct-to-consumer strategies, but now the field sees mergers and business-to-business models. Only Singapore, Israel, and the United States have given regulatory approval for cell-based meat, and the UK approved it for pet food, requiring further regulatory success globally. Efforts to scale cultured biomass for commercial production have yet to achieve economic viability [9], leading to "hybrid" foods blending animal and plant materials. In principle, three types of biological input materials might be required, (i.) the desired cells of the target organism (Fig. 1A), (ii.) additional plant materials for the creation of bio-scaffolds (Fig. 1B), and (iii.) microorganisms that might be involved with the processing of media to nourish the target cells (Fig. 1C). This paper focuses on early stages of CellAg, emphasizing procurement of one of the biological input materials, animal cells.

**Fig. 1:**
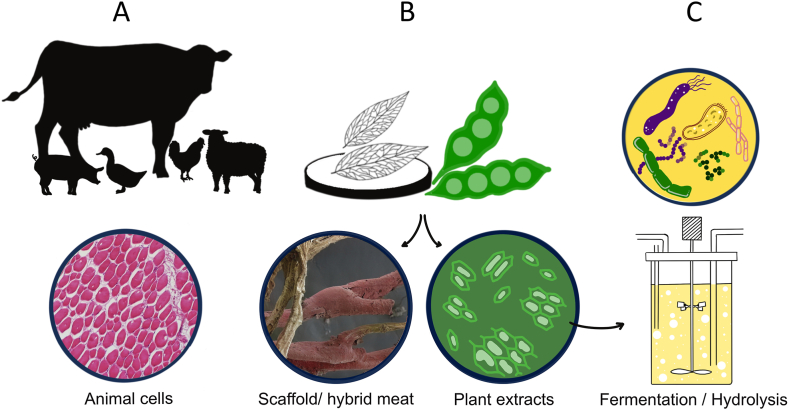
Source organisms that can contribute to the cultured meat product. Source organisms from different biological domains such as animals, plants and microbes can contribute to the production of cultured meat products by providing initial materials like cells, media components, and bio-scaffolds. Healthy animals can be used to provide cells required for creating cultured meat products (A). Cells can be grown in media that utilize refined plant extracts (B) and/or fermented or hydrolysed plant materials (C) as a media component to support their proliferation, thus reducing serum dependency. Various species can be used diversly to get the desired structure, such as decellularized leaves being used as a bio-scaffold, to create a cultured meat product [56].

### 1.3 Reverse engineering of consumer preference

Consumer acceptance is crucial for the success of cultured meat, regardless of technical feasibility or sustainability [10]. Unlike conventional meat, CellAg allows precise control over product characteristics, starting with the development of source cell lines from acceptable organisms to ensure market adoption. While substitutes like microalgae, fungi, yeast, and insects have been used for years [11,12], CellAg products must address overall perception of the consumer about safety, GMOs, etc.

Theoretically, CellAg products have the capacity to provide a range of advantages to society, particularly when leveraging recent advancements such as, closed-loop methods that allow media recycling [13], using decellularised scaffolds from agricultural waste [14], using 3D porous gelatin micro-carriers [15], and more advancements in bioreactor and 3D printing technologies [16]. Along with researchers’ focus on taste and texture, policy makers and companies should also educate consumers about the consistent quality and potential benefits of cultured compared to traditional meat types [17]. As CellAg products become available, public discussions will address input materials, such as GMOs and embryonal or pluripotent stem cells, or any recombinant cultured materials, which may classify cultured meat as 'Novel foods' (i.e., food derived from cell or tissue culture sourced from animals, plants, fungi, or microorganisms) or ultra-processed, under current regulations [18]. Consumer demand for tasty, affordable, and convenient foods with favourable environmental, social and governance (ESG) properties will influence the acceptance of cultured meat.

### 1.4 Requirements for input materials

Creating cultured meat requires three key initial input materials, animal cells, media and scaffolding material. Optimizing cell culture media is crucial for cultured meat production, with significant efforts from corporations and academia. Dutch company Mosa Meat B.V. reduced cost by 88 % through refining media components and avoiding fetal bovine serum (FBS), which poses issues due to its animal origin and batch variability [19,20]. Serum-free supplements and chemically defined substitutes, like Essential 8™, TeSR™, and FBM™, have been developed [[21], [22], [23]], but matching the effectiveness of serum-based media, particularly for livestock cells, remains challenging [24,25]. Recombinant proteins and cost-effective mimics are being explored to further reduce costs as well [26,27]. Optimizing scaffolding materials is also essential for achieving the desired texture in cultured meat, effective scaffolds like 3D printed hydrogels, algae-based scaffolds, and nanocellulose are being developed [[28], [29], [30]]. These scaffolds must be edible, support cell adherence, and be cost-effective. Options include animal-derived (gelatin, collagen), plant-derived (polysaccharides), and synthetic materials, each with unique benefits and challenges [[31], [32], [33], [34]]. Selecting the right scaffold is crucial for developing marketable cultured meat products.

As for the third key input material, animal cells, meat comprises approximately 90 % muscle fibres, 10 % fat and connective tissue, and less than 1 % blood [35,36]. For cultivated meat, the primary components are skeletal myocytes and adipocytes, with fibroblasts, chondrocytes, and hematopoietic cells playing supporting roles. Common available cell lines used for biomedical and fundamental research include the C2C12 myoblasts from mice and QM7 from Japanese quail [37,38]. Adult stem cells, such as muscle satellite cells, mesenchymal stem cells (MSCs), and fibro/adipogenic progenitors (FAPs), are crucial for differentiation into mature tissue types. MSCs, sourced from bone marrow and other sites, can become adipocytes, chondrocytes, and fibroblasts, while FAPs support muscle tissue development [39,40]. Pluripotent stem cells, including embryonic stem cells (ESCs) and induced pluripotent stem cells (iPSCs), offer high proliferation and differentiation potential but face challenges in protocol establishment and ethical considerations [41,42]. Direct reprogramming of somatic cells into muscle progenitors is another promising, yet only developing, approach [43].

The primary component of meat is skeletal muscle cells, however, differentiated skeletal muscle cells are not suitable for *in vitro* expansion as they lose their proliferative ability under normal conditions. Therefore, the logical starting point is to isolate myosatellite muscle cells. They can differentiate into mature muscle tissue by altering media conditions [44]. For the growth of these cells once isolated, functional proteins are required, which can be obtained from FBS or specific growth factors [45] or heme proteins [46]. Within this paper, we have established a methodology for isolating bovine myofibroblast cells, which were spontaneously immortalized and, characterised through RNA sequencing and western blotting.

## 2 Methods

Unless specified otherwise, all chemicals were obtained from Merck KGaA (Darmstadt, Germany).

*Myofibroblast cell isolation from bovine tissue*: Bovine muscle tissue was acquired from the exterior hind leg of a Black Angus domesticated cow (*Bos taurus*) immediately post-slaughter by a mobile butcher unit on a beef farm located in Berry (NSW, 2535, Australia). No modification had been made to alter usual procedures. Immediately after the receipt of muscle tissue, it was briefly cleaned with isotonic saline and placed on ice in DMEM supplemented with Penicillin (10 U/mL), Streptomycin (10 mg/mL) and Amphotercin B (2.5 mg/mL) for transport to university laboratories. Muscle tissue was processed immediately using an adapted protocol by Simsa and colleagues [46]. Following removal of any non-muscle tissue, ∼1 g of muscle was briefly minced using a scalpel, washed with excess DMEM and then placed into a gentleMACS C-tube in DMEM containing 0.1 % w/v collagenase II (Type II) and digested to a form a cell suspension using a gentleMACS Octo Dissociator at 37 °C (Miltenyi Biotec). FBS was added to the cell suspension (20 % v/v) and the digest was filtered sequentially through 100-, 70- and 40-mm cell strainers. Cells were then pelleted by centrifugation at 200*g* for 5 min. The cell pellet was washed once with DMEM and then resuspended in complete growth media (DMEM supplemented with 20 % v/v FBS and 1 ng/mL FGF-Basic and antibiotics - Penicillin (10 U/mL); Streptomycin (10 mg/mL); Primocin (100 mg/mL; Invivogen CA, USA) and titrated in 96 well plates and cells were permitted to attach overnight (37 °C/5 % CO_2_). Wells that were visually confirmed to contain single colonies were expanded and used in this study (putative fibroblast fraction). Non-attached cells (putative myosatellite fraction) were re-attached on laminin coated wells and expanded but were not used in this study.

*Histopathology of Bovine tissues:* Along with bovine muscle tissue, different organ tissues were collected for histopathology staining. These were fixed in 10 % phosphate buffered formalin during transportation. Followed by staining, using Hematoxylin and eosin (H&E) stain and observed under light microscope at the School of Clinical Medicine, University of New South Wales, Sydney, Australia.

*Cell expansion and passaging using live-cell imaging:* Expanded bovine cells were plated at 2.5 × 10^5^ cells per well in a 6-well plate in triplicate and growth was monitored by phase contrast microscopy using an Incucyte S3 Live-Cell Analysis Instrument (Sartorius, Göttingen, Germany) for 100 days/30 passages. During the expansion phase the FBS concentration was lowered to 10 % v/v. Phase images were acquired every 12h and densitometry of confluence was measured in these images by the Incucyte software with a segmentation adjustment 1.2 and minimum area filter of 800 mm^2^. At the point of manuscript submission these bovine myofibroblasts (*Bov.mia*) are still dividing with a similar doubling time (∼200 days in culture).

*Doubling time calculation*: The doubling time of cells was determined using the standard formula: doubling time = duration.ln(2) = duration x ln(2)/ln(final confluence/initial confluence), where the confluence value was determined using the Incucyte S3 Live-Cell Analysis Instrument software with the above listed settings.

*Transcriptomics analysis of cellular material using RNA Sequencing:* Total RNA was extracted from each myofibroblast isolated from two different muscle tissues using the TRIzol reagent (Invitrogen, USA) and following manufacturer's instructions. The quality of RNA samples was estimated using a UV–vis spectrophotometer NanoDrop ND-1000 (NanoDrop Technologies, Wilmington, DE). RNA integrity was quantified at Ramaciotti Centre for Genomics (UNSW Sydney, Australia), by running each sample on an RNA chip in an Agilent TapeStation (Agilent Technologies, Palo Alto, CA, USA). The software analysed the entire electrophoretic trace including the ribosomal RNA peaks and determined an RNA integrity number (RIN). The RIN was used as a measure to optimize the interpretation of RNA quality. In the present study, a conventional threshold (RIN number ≥7.0; 260/280 and 260/230 ratios to between 1.8 and 2.2) was set to decrease the experimental biases due to poor RNA quality. RNA-seq was conducted on an Illumina NextSeq 1000 System by Ramaciotti Centre for Genomics (UNSW Sydney, Australia).

The RNA-seq reads were quality checked using FASTQC, a quality control tool for high throughput sequencing data (Babraham Institute in Cambridge) and seqmonk (Babraham Institute in Cambridge). The expression of transcripts was quantified using Salmon by mapping and aligning with the bovine reference genome sequence (ARS-UCD1.3) [47]. The raw transcriptomic counts were normalised using Transcripts per million (TPM) units for gene expression analysis.

To confirm reads were from Bovine origin/source, the reads were taxonomically classified using Kraken2 against the kraken2 standard database (containing archaea, bacteria, viral and human genomes) and a custom database containing bovine, human and mouse reference genome [48]. After confirming the bovine source, canonical markers specific for myofibroblast cell type were matched using PanglaoDB database [49]. A reference study on transcriptomic profiling was also used to confirm the mRNAs associated with myofibroblasts [50]. After confirming source and cell type, five markers were selected to further confirm the cell type using protein analysis by western blotting.

*Proteomics analysis of cellular material using Western Blot analysis:* Following removal of growth media, cell monolayers were washed once with ice cold PBS, pH 7.2, 1 mM EDTA. RIPA buffer (25 mM Tris-HCl pH 7.6, 150 mm NaCl, 5 mM EDTA, 1 % v/v IGEPAL CA-630, 1 % w/v sodium deoxycholate, 0.1 % w/v SDS containing protease inhibitors) was added directly to washed sub-confluent monolayers of bovine cells at a ratio of ∼ 1 mL RIPA per 150 cm^2^ of cell culture plastic. Lysed cellular material was scraped, kept on ice, and sonicated using the microtip of a Branson 250 Analog Sonifier (Branson Ultrasonics, CT, USA; output control set to “1”, 50 % duty cycle, 20 pulses). The cell lysate was clarified by centrifugation (10,000×*g*) and then subjected to Western blot analysis as follows. Clarified cell lysate was subjected to SDS-PAGE on a 4–20 % acrylamide gel on a Mini-PROTEAN Electrophoresis cell (Bio-Rad Laboratories, CA, USA). Electrophoretically separated proteins were transferred to Immobilon-P PVDF membrane (25V/RT/overnight). Membranes were blocked (5 % w/v BSA in 10 mM Tris (pH 7.6), 150 mM NaCl containing 0.05 % v/v TWEEN20 detergent (TBS-T).

Membranes were incubated overnight at 4 °C in blocker with the following monoclonal antibodies: a-smooth muscle actin (clone D4K9N), Vimentin (clone D21H3), Fibroblast activation protein (FAP; clone E1V9V), S100A4 (clone D9F9D), all from Cell Signaling Technology, MA, USA, or loading control, anti-b-actin (clone AC-15 from Merck). This was followed by incubation with secondary anti-rabbit IgG HRP linked secondary antibody (Cell Signaling Technology, MA, USA). Antibodies used in this study were not validated against a bovine protein source at the point of purchase. To source antibodies that had a high chance of bovine reactivity we performed protein sequence alignments between the known antibody recognition species (human) and the target species (bovine). We used relevant protein targets that had >90 % sequence identity between bovine and human sequences. Where additional antigenic information of the antibody was made available by the manufacturer (e.g. C-terminal, N-terminal, or a specific amino acid sequence), then alignments were performed on that individual sequence area alone. Chemiluminescent bands were detected using the SignalFire™ ECL substrate system (Cell Signaling Technology) with images acquired on a ChemiDoc Imaging System (Bio-Rad).

## 3 Results

*Histopathology:* Histopathology staining of the collected organ and muscle tissues was employed as a routine step for validation of general donor health, and no apparent pathologies had been detected. The health status of the animal is supported by the analysis of organ tissue, as shown in Fig. 2(A–D) & 3 (see Fig. 3).

**Fig. 2:**
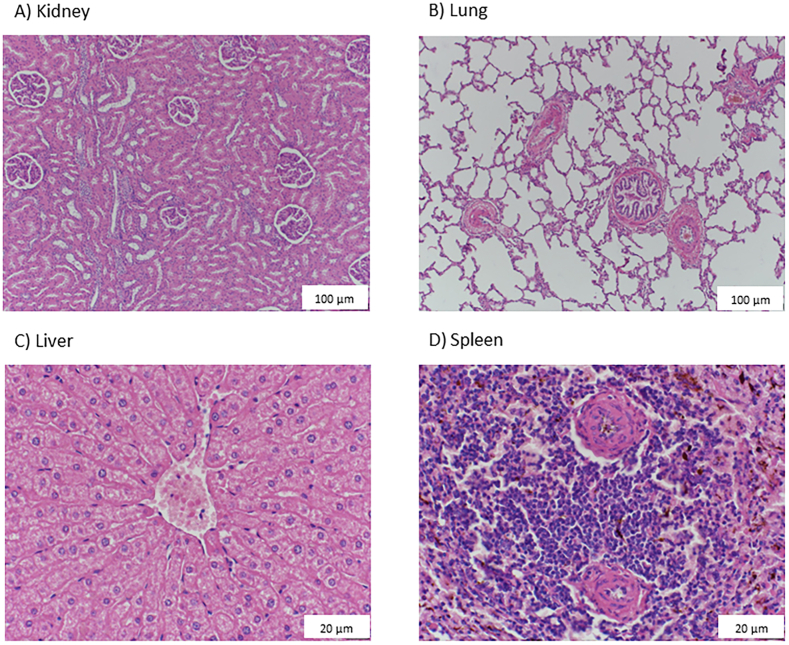
Histopathology images of bovine organ tissues to validate health status during tissue collection. Bovine organ tissue samples were collected and subjected to histopathology staining to establish a normal and healthy animal. (A) Kidney, longitudinal section; (B) lung, longitudinal section; (C) Liver, longitudinal section; (D) Spleen longitudinal section. Scale bar, 20–100 μm.

**Fig. 3:**
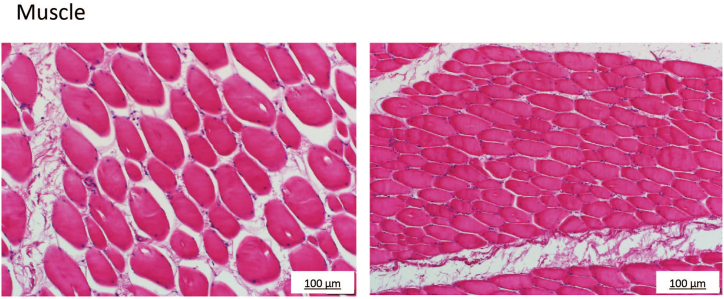
Histopathology images of bovine muscle tissue prior to cell line development. Skeletal muscle cross sectional and longitudinal. Bovine muscle tissues collected as a source were subjected to histopathology staining to validate the source for cellular isolation and as an additional sign of a healthy animal. Bovine tissue was collected in Berry, NSW, Australia. Scale bar, 100 μm.

*Growth of fibroblasts*: The growth profile of the bovine myofibroblast cells was monitored by phase contrast microscopy using an Incucyte S3 Live cell imager (Fig. 4). Over a period of 100 days, this procedure included 30 passages. The doubling time of the cells throughout the 100-day observation period is presented in Fig. 4E, and the percentage of confluence observed through phase contrast microscopy is shown in Fig. 4D. During this period, the variation in doubling was observed until approximately 55 days in the monitored culture. At this point, the cells maintained a consistent doubling time of around 36 h, which remained consistent until ∼100 days in culture.

**Fig. 4:**
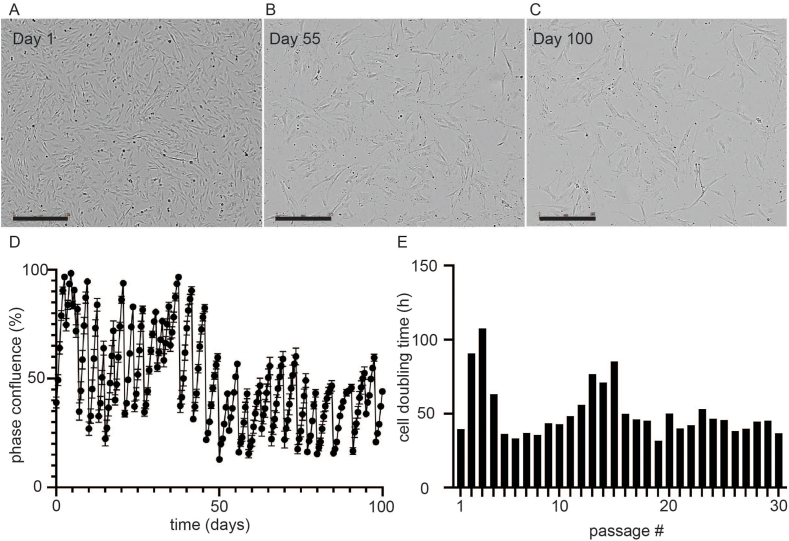
**Stable growth of cells from fibroblast fraction of bovine muscle tissue.** (A–C) Phase contrast images of the isolated cells following (A) 1, (B) 55 or (C) 100 days of culture within an Incucyte S3 live cell imaging instrument. Scale bars = 400 μm. (D) Degree of cell confluence over 100 days of cell culture and 30 independent passages of the myofibroblasts. The degree of cell confluence, “phase confluence (%)”, was determined by the internal software of an Incucyte S3 live cell imager which performs densitometry on phase contrast images acquired over the 100-day time course. Cell doubling time over 30 independent passages, as calculated by the formula: doubling time = duration.ln(2) = duration x ln(2)/ln(final confluence/initial confluence).

A comparison of the physical appearance displayed by the cells under phase contrast microscopy at days 1, 55 and 100 in the monitored culture is illustrated in Fig. 4 (A, B, and C). It can be observed that throughout the 100 days, cells exhibited a fibroblast-like morphology. However, from Day 55 onwards, the cells were noticeably larger. This visible phenotype observed from Day 55 remained stable and was consistently seen through to the 100 days in culture (Fig. 4A, B and C).

*Summary of RNA-Seq analysis*: Total RNA was extracted from the isolated bovine myofibroblasts using the TRIzol reagent, and the quality of RNA was assessed, yielding measurements of at 1.88 (260:280) and 0.95 (260:230). RNA sequencing of the *Bov.mia* myofibroblasts generated approximately 43 million raw single-end sequence reads. Following a quality check using FASTQC, the reads were successfully mapped to the *Bos taurus* NCBI genome assembly (ARS-UCD1.3) as described by Patro et al. [47], The mapping results indicated that an average of 97.5 % of the reads aligned across all three different databases - Kraken2, Hisat2 and seqmonk, thus confirming the bovine origin of the cells.

This confirmation of species origin was followed by confirmation of cell type by identifying the canonical markers for the myofibroblast cell type. Among the abundantly expressed genes with the highest TPM counts, *actin alpha 2- smooth muscle* (*ACTA2 or a-SMA*), *caledesmon 1* (*CALD1*), *myosin light chain 9* (*MYL9*), and *transgelin* (*TAGLN*) were identified as the associated markers for myofibroblast cell type (Table 1). A study by Ramalingam et al. identified 30 differentially expressed genes (DEGs) associated to bovine fibroblasts [50]. They also linked these DEGs to 10 significantly enriched gene ontology (GO) terms, all of which play a crucial role in muscle growth, development, and collagen synthesis. The analysis presented here finds these 30 DEGs to be expressed in our myofibroblasts. Additionally, 3 out of 4 markers that were selected for proteomic analyses were observed as abundantly expressed in RNA sequencing.

**Table 1:** Differential mRNAs associated with myofibroblast.

Reference	Gene symbol	Gene name	Bov.mia TRP
**Canonical markers for myofibroblast** [49]	*ACTA2*	*actin alpha 2, smooth muscle*	5212.44
	*CALD1*	*caldesmon 1*	1450.01
	*MYL9*	*myosin light chain 9*	820.87
	*TAGLN*	*transgelin*	772.34
**Reference Markers** [50]	*COL1A1*	*collagen type I alpha 1 chain*	19271.80
	*VIM*	*vimentin*	6741.03
	*ACTA2*	*actin alpha 2, smooth muscle*	5212.44
	*COL3A1*	*collagen type III alpha 1 chain*	3676.76
	*LOX*	*lysyl oxidase*	1519.37
	*S100A4*	*S100 calcium binding protein A4*	1508.11
	*MYH9*	*myosin heavy chain 9*	1152.68
	*HSP90B1*	*heat shock protein 90 beta family member 1*	1046.48
	*HSPA5*	*heat shock protein family A (Hsp70) member 5*	984.89
	*HSPA8*	*heat shock protein family A (Hsp70) member 8*	903.22
	*HSPA9*	*heat shock protein family A (Hsp70) member 9*	350.29
	*CAPN2*	*calpain 2*	280.03
	*HSPA1A*	*heat shock protein family A (Hsp70) member 1A*	150.89
	*CAPN1*	*calpain 1*	112.24
	*HSPA4*	*heat shock protein family A (Hsp70) member 4*	109.86
	*BMP1*	*bone morphogenetic protein 1*	86.00
	*FAP*	*fibroblast activation protein alpha*	45.34
	*PDGFA*	*platelet derived growth factor subunit A*	21.21
	*DGAT1*	*diacylglycerol O-acyltransferase 1*	14.45
	*CASP3*	*caspase 3*	8.83
	*PPARG*	*peroxisome proliferator activated receptor gamma*	2.49
**Selected markers for WB**	*VIM*	*vimentin*	6741.03
	*ACTA2*	*actin alpha 2, smooth muscle*	5212.44
	*S100A4*	*S100 calcium binding protein A4*	1508.11
	*FAP*	*fibroblast activation protein alpha*	45.34

The table shows genes that are canonical markers for myofibroblast cell type using Panglao database and from Ramalingam et al. Additionally, the table shows selected and confirmed markers through protein analysis. TRP, transcript per million; WB, western blotting.

*Summary of proteomic analyses*: Given that the cells are derived from the fibroblast fraction of the cell isolation process, we performed Western blot analysis using total cell lysates generated from cells cultured for approximately 100 days. We selected 3 markers, which were abundantly expressed in our RNAseq analysis, vimentin (VIM), actin alpha 2- smooth muscle (ACTA2 or a-SMA), S100 calcium binding protein A4 (S100A4). Along with a specific myofibroblast marker, fibroblast activation protein (FAP). The protein expression levels of myofibroblast markers were detected in these total cell lysates (Fig. 5A–D). Collectively, this expression profile corresponds to a myofibroblast cell phenotype.

**Fig. 5:**
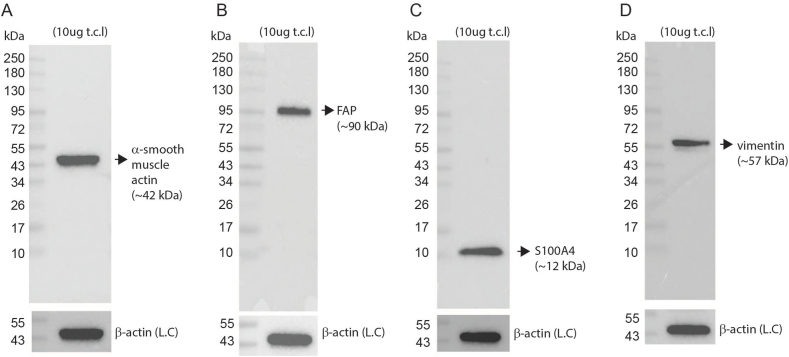
The cell line isolated from the fibroblast fraction of bovine muscle tissue expressed protein markers consistent with a myofibroblast phenotype. (A-D) Western blot analysis of total cellular lysates (“tcl”) with antibodies directed at myofibroblast markers. (A) α-smooth muscle actin, (B) Fibroblast activation protein/seprase (FAP), (C) S100A4, or (D) vimentin. (A-D) β-actin is shown as a sample loading control (L.C.). Original images for A-D can be found in the supplementary section.

## 4 Discussion

Procurement of healthy cell-based material that can develop into a nutrient dense source for human consumption is a critical challenge for the emerging CellAg industry. The developments in this field over the past few years demonstrate significant issues surrounding the ramping up of this technology to reach a broader consumer segment and to achieve an economy of scale for production of these new materials. Simply boosting the amount of cell-derived biomass that is being created will be important but not sufficient because consumers might broadly reject the products proposed and ultimately, they might reject the technology and the CellAg industry as a whole.

The problems encountered in the field are linked with the very nature of cell growth and with taking cells out of the animal to grow them *in vitro*. Typically, primary cells have a limitation in growth before they enter senescence determined by their proliferative capacity that is limited by the Hayflick number of about 50 doublings and practically often less than that. It is for this limit in proliferative capacity that cell lines by definition should be immortalized. There have been recent advancements in the spontaneous immortalization of cells, which do not rely on genome editing. While there are benefits of genome editing, such as e.g.faster results, they would gain limited or no consumer acceptance. On the other hand, natural immortalization allows cells to proliferate indefinitely without genetic modification, benefiting CellAg technology. There have been key advancements including understanding molecular pathways [51,52], selecting optimal cell types like fibroblasts [53], etc. Immortalization also addresses ethical concerns and integrates well with bioreactor technologies, enhancing cultured meat scalability and commercialization potential. These innovations offer a path to sustainable, ethically acceptable cultured meat products, impacting food security and environmental sustainability. FBS is crucial during the initial stages of cell isolation, particularly for the first few passages, before adapting the cells to a serum-free medium. While we recognize that the cells described here currently require serum, the next step is to adapt them to a serum-free medium. Recent work indicates that plant-based hydrolysates might serve well as building blocks for serum free media preparations [54].

The bovine muscle tissue sourced for this study was processed using an adapted protocol of Kaplan and colleagues [46]. From this cell isolation technique, we procured both myofibroblasts and myoblasts that could form myotubes in culture. Only the myofibroblast line (*Bov.mia*) was used in the current study due to their more stable doubling time over more extended periods of the first 100 days in culture. We concluded the cell line is a myofibroblast phenotype by both protein and RNAseq analyses. The stable doubling time of the cell line of ∼36 h is far from ideal, but it is a possibility that this will decrease once the line spontaneously immortalizes.

With the approach presented here, no IPSC material is being used or developed and equally no transgenic material is developed that could be classified as GMO food. Natural immortalization of cell lines appears the gentlest way forward to address the requirements of the CellAg industry and the principles described do not only apply to driving the development of long-lasting cell lines but also the development of high performing, robust and proliferative cell lines.

The consumer discussion around novel food material is not always rational, and while GMO food might be innocuous from a toxicological perspective, it will certainly give rise to criticism, if not attack, from consumer groups. The key metric for a successful CellAg industry will be the ability to provide food, that along with the added environmental and ethical benefits, is produced in a sufficient, safe, and nutritious manner, i.e., with the ability to produce sufficient biomass. Meeting this objective will lead to considering CellAg as a relevant option [55].

By creating an immortalized fibroblast line, *Bov.mia*, capable of unlimited proliferation while retaining the traits of parent tissue cells, we streamlined the labor-intensive process of isolating primary cells. This will reduce researchers' time, energy, and help cut experimental costs. It will also facilitate scientific inquiries such as livestock gene function, accelerating scientific progress. However, the standardization of immortalization methods across species and ensuring the preservation of original cell traits in immortalized cells remain unresolved challenges necessitating further investigation. Despite these challenges, the application of immortalized cells holds vast potential.

## 5 Conclusion

Bovine myofibroblasts isolated directly from muscle tissue can act as a principal source of input material for the creation of immortalized cell lines suitable for expansion and consumption. In this study we present spontaneous immortalization of a stable myofibroblast cell line that was characterised through RNA sequencing and proteomics. These cells are natural and suitable for lab-scale development in CellAg.

## Funding

This work has been made possible with the support of an Academic Startup Funds (ASUF) provided by 10.13039/501100001773UNSW, Sydney to Prof. Johannes le Coutre.

## Data availability statement

Data included in article/supplementary material is referenced in the article.

## CRediT authorship contribution statement

**Apeksha Bharatgiri Goswami:** Writing – review & editing, Writing – original draft, Investigation, Formal analysis, Data curation, Conceptualization. **Mark S. Rybchyn:** Writing – review & editing, Writing – original draft, Validation, Methodology, Investigation, Formal analysis, Data curation, Conceptualization. **W.R. Walsh:** Methodology, Investigation, Formal analysis, Data curation. **Johannes le Coutre:** Writing – review & editing, Writing – original draft, Validation, Supervision, Resources, Project administration, Methodology, Investigation, Funding acquisition, Data curation, Conceptualization.

## Declaration of competing interest

The authors declare the following financial interests/personal relationships which may be considered as potential competing interests:Johannes le Coutre reports a relationship with University of New South Wales that includes: employment. If there are other authors, they declare that they have no known competing financial interests or personal relationships that could have appeared to influence the work reported in this paper.

## Figures and Tables

**figs1:**
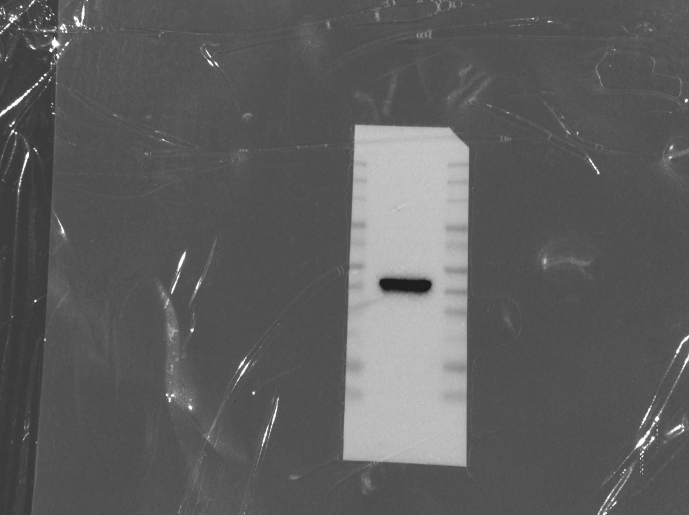


**figs2:**
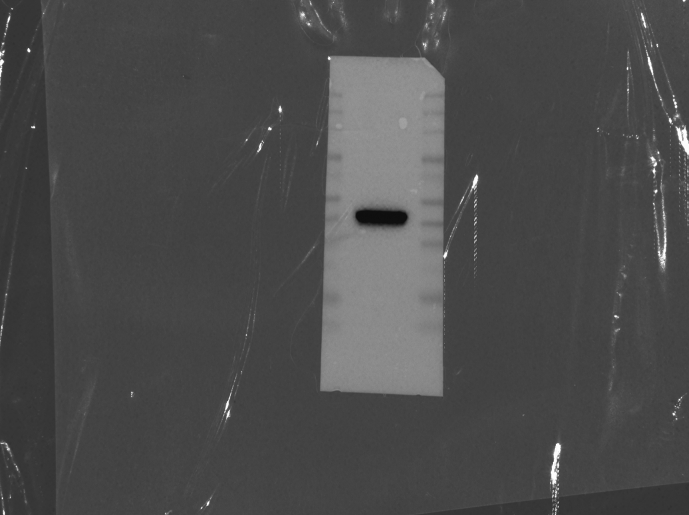


**figs3:**
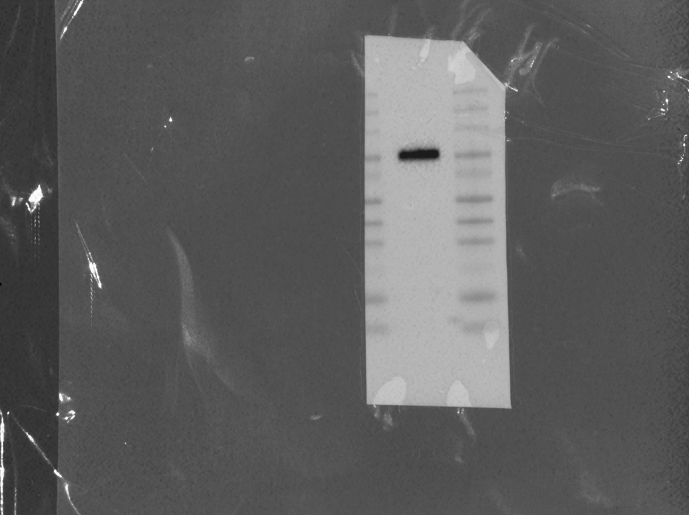


**figs4:**
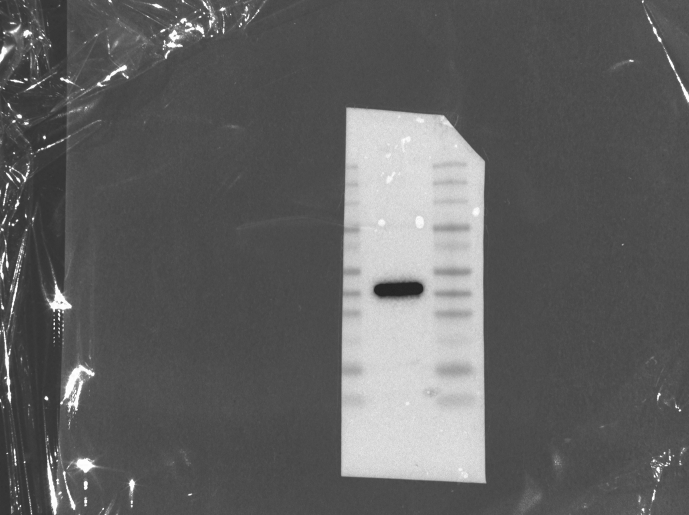


**figs5:**
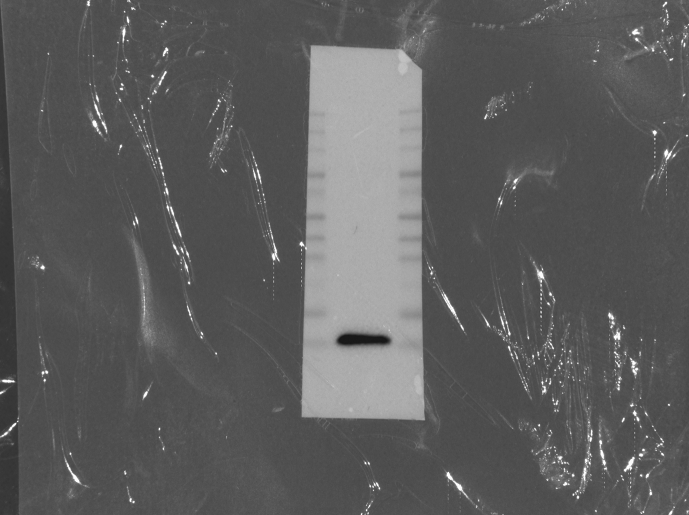


**figs6:**
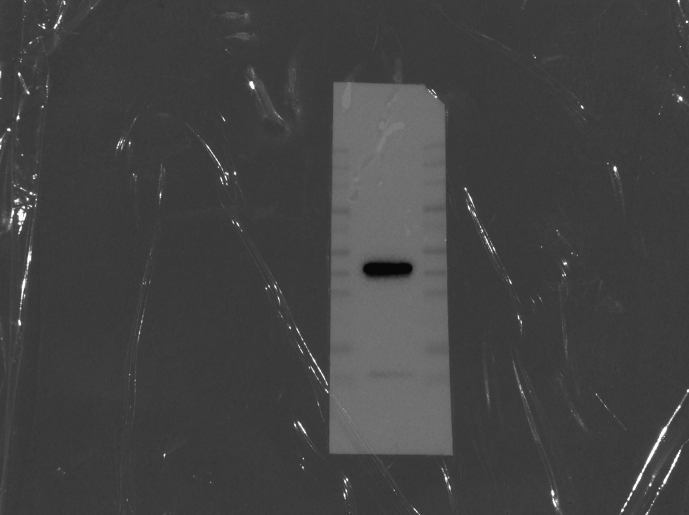


**figs7:**
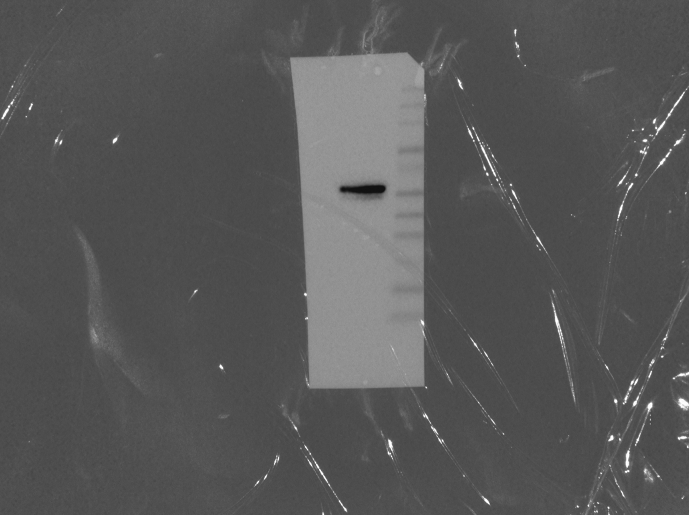


**figs8:**